# Case Report: Ovarian endometrioid tumor occurring in a patient with early-stage, low-risk endometrial carcinoma successfully treated by fertility-sparing management

**DOI:** 10.3389/fonc.2025.1696640

**Published:** 2025-10-07

**Authors:** Shuxu Tian, Huangbei Song, Fang Wang, Juan Hao, Xuan Liu, Chenchen Geng

**Affiliations:** ^1^ Department of Gynecology, Qingdao Women and Children’s Hospital, Qingdao University, Qingdao, China; ^2^ Department of Pathology, Qingdao Women and Children’s Hospital, Qingdao University, Qingdao, China; ^3^ Department of Ultrasound, Qilu Hospital of Shandong University (Qingdao), Qingdao, China

**Keywords:** endometrial carcinoma, fertility-sparing, levonorgestrel-releasing intrauterine system, ovarian tumor, case report

## Abstract

**Rationale:**

Endometrial carcinoma (EC) increasingly affects younger women, prompting interest in fertility-sparing treatments. Although hormonal therapy is a feasible option for carefully selected patients, there remains a substantial risk of recurrence or associated ovarian malignancy.

**Case presentation:**

A 35-year-old premenopausal woman reported abnormal uterine bleeding characterized by increased menstrual flow over approximately one year.

**Diagnosis and intervention:**

She was diagnosed with stage IA, grade 1 EC managed initially with high-dose oral megestrol acetate followed by a levonorgestrel-releasing intrauterine system due to intolerance. Serial endometrial biopsies demonstrated histologic remission, after which the patient elected definitive surgery with hysterectomy and bilateral salpingectomy while preserving the ovaries. Uterine pathology confirmed absence of residual carcinoma.

**Follow-up and outcomes:**

During follow-up, a right adnexal cystic-solid mass was detected and categorized as Ovarian-Adnexal Reporting and Data System (O-RADS) ultrasound category 4, with MRI features raising suspicion. Comprehensive surgical staging confirmed a unilateral low-grade ovarian endometrioid carcinoma with squamous differentiation; staging and peritoneal cytology were negative. The patient recovered uneventfully and remains under surveillance without adjuvant therapy.

**Lessons:**

This case highlights the rare occurrence of metachronous ovarian endometrioid carcinoma after successful fertility-sparing and hysterectomy, underscores the importance of shared decision-making regarding ovarian preservation, and supports risk-adapted surveillance strategies in this population.

## 1 Introduction

Endometrial carcinoma (EC) is the most common gynecologic malignancy in many high-income countries, with rising global incidence and mortality. Although most cases occur postmenopausally, 4.2% present in women under 40 years, a proportion that appears to be increasing alongside obesity and metabolic risk factors (1). Young women with low-risk, early-stage endometrial carcinoma (stage IA, grade 1), particularly those desiring fertility preservation, often choose conservative management involving hormonal progestin therapy. Conservative management is generally reserved for strictly selected patients with FIGO stage IA, grade 1 endometrioid carcinoma without myometrial invasion or extrauterine disease, after thorough imaging to exclude adnexal and nodal involvement. Contemporary guidelines further recommend mismatch repair (MMR) assessment and, where available, molecular profiling (e.g., POLE and p53 status) to refine risk and exclude high-risk biology before initiating fertility-sparing therapy. While progestins can successfully induce remission, risks of disease recurrence, progression, and subsequent ovarian malignancies remain important considerations following conservative treatment. Here, we present a case of a 35-year-old patient, who despite achieving histological remission through successful fertility-sparing therapy and subsequent hysterectomy, developed an ovarian endometrioid carcinoma during follow-up.

## 2 Case report

### 2.1 Case description

A 35-year-old premenopausal woman of Chinese ethnicity presented for evaluation. She has had one previous full-term vaginal delivery. Relevant medical, surgical, and psychosocial histories were reviewed; there were no notable comorbidities or prior gynecologic surgeries; family history of malignancy was unremarkable. Body mass index was 19.57 kg/m^2^. There was no record of prior genetic testing. She reported abnormal uterine bleeding characterized by increased menstrual flow over approximately one year, without intermenstrual or postcoital bleeding. She was not using hormonal contraception at presentation. She denied over-the-counter or herbal remedy use. Cervical screening status was negative. Physical examination was unremarkable. Transvaginal ultrasound demonstrated heterogeneous endometrial echotexture with an endometrial thickness of approximately 14 mm. Baseline tumor markers (CA-125 10.2 U/mL; HE4 36.5 pmol/L) were within normal limits.

### 2.2 Diagnosis and intervention

Diagnostic curettage revealed atypical hyperplasia transitioning focally into grade 1 endometrioid carcinoma with squamous differentiation and wild-type p53 expression. Pelvic MRI indicated stage IA EC confined to the endometrium without myometrial invasion or extrauterine abnormalities. Given her desire to preserve fertility, the patient received comprehensive counseling on standard versus fertility-sparing options, including oncologic risks, response rates, surveillance requirements, and the implications of ovarian conservation. She elected conservative therapy and initiated oral megestrol acetate (MA) 160 mg twice daily (total 320 mg/day). Follow-up hysteroscopy after two months showed thin endometrium; biopsy demonstrated secretory transformation signifying a promising response. Oral MA therapy was subsequently discontinued due to significant gastrointestinal side effects, replaced by a levonorgestrel-releasing intrauterine system (LNG-IUS). Serial endometrial biopsies at approximately 3–6 month intervals and again at 6 months and 12 months demonstrated histologic remission without atypia or carcinoma. After removal of the LNG-IUS at 13 months, the patient was advised to attempt expedited pregnancy or consider luteal-phase progestin therapy. However, her reproductive goals subsequently changed, and at 18 months, after thorough counseling, she underwent laparoscopic total hysterectomy with bilateral salpingectomy, preserving both ovaries due to patient preference and absence of abnormal findings. Surgical histopathology indicated secretory-phase endometrium without residual carcinoma. Regular postoperative ultrasonography and pelvic exam every 3–6 months initially showed normal ovaries.

### 2.3 Follow-up and outcomes

**Figure 1:**
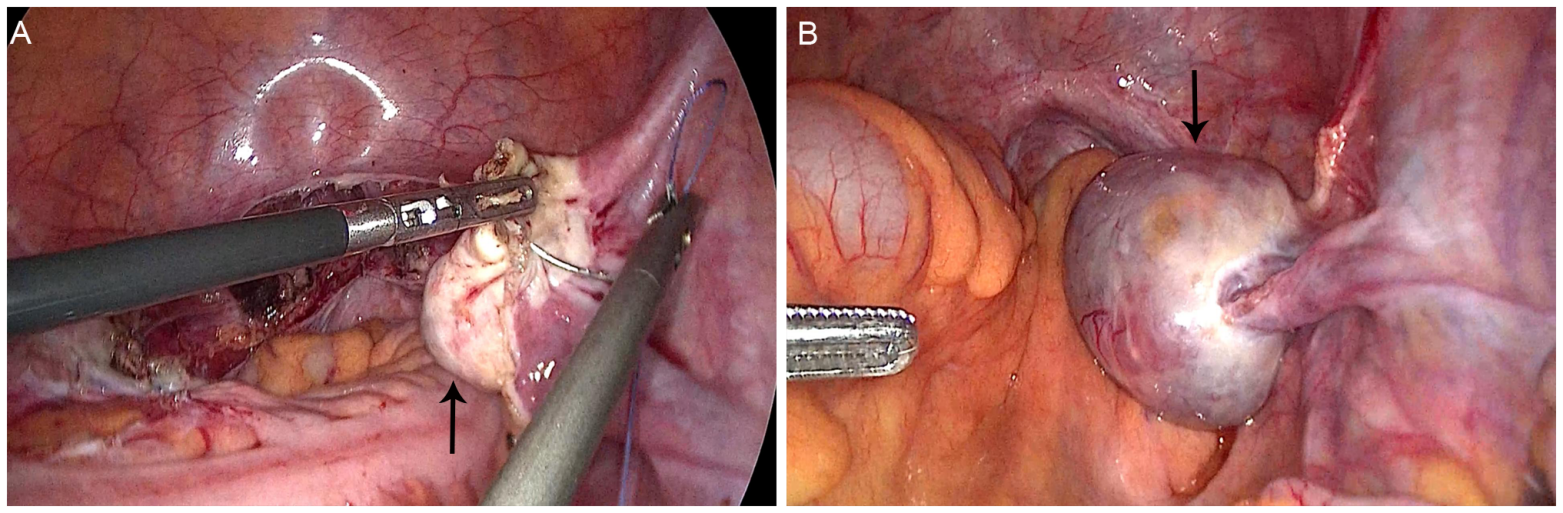
Morphology of the right ovary during two laparoscopic surgeries. **(A)** Normal appearance of the right ovary in the first surgery (arrow); **(B)** Enlarged right ovary with a cystic-solid tumor in the second surgery (arrow).

**Figure 2:**
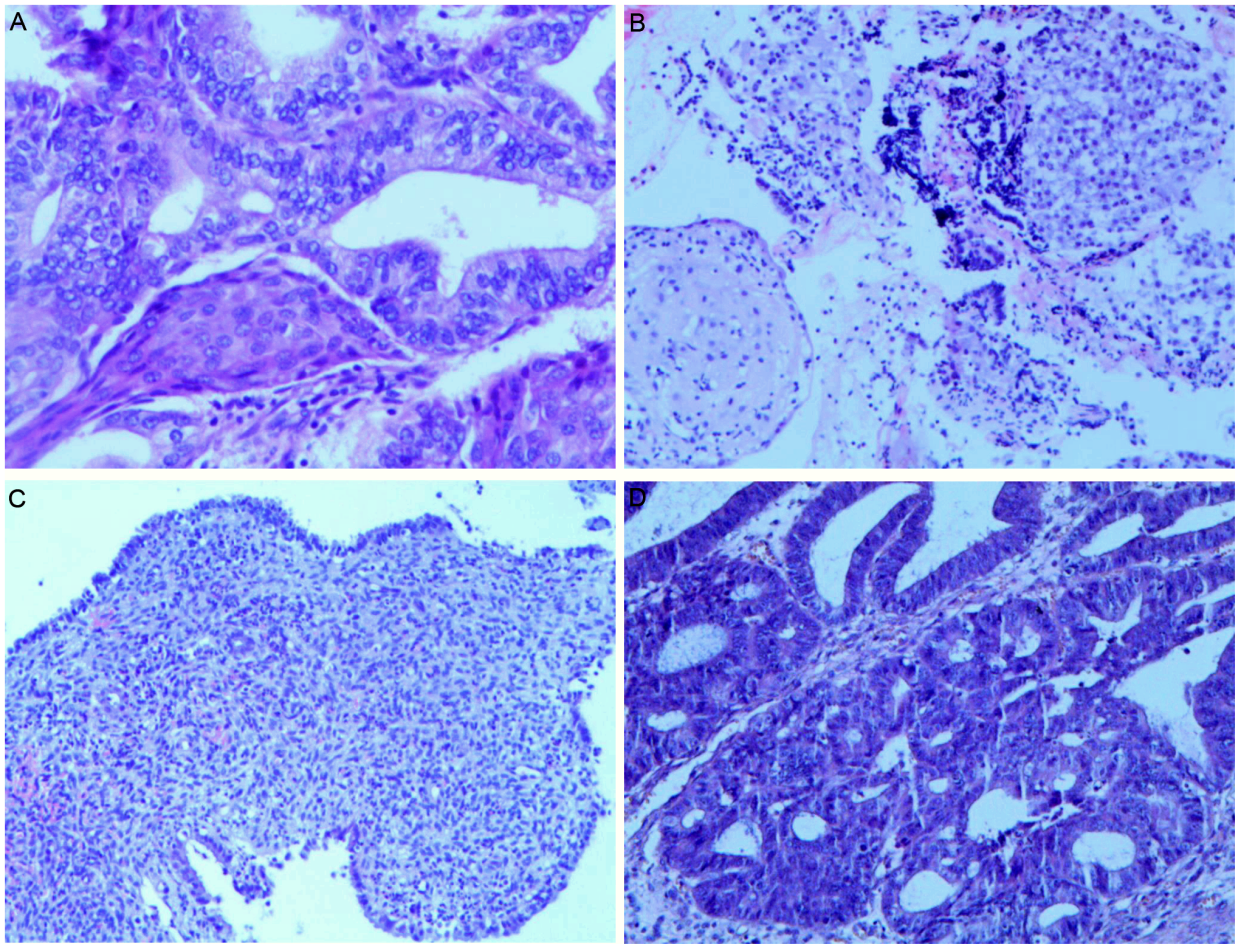
Pathological changes of endometrium during the treatment and the right ovarian tumor. **(A)** Grade 1 endometrioid carcinoma with squamous differentiation before fertility-sparing treatment (magnification: ×200); **(B)** Endometrium after progestins treatment, with partial stromal decidualization (magnification: ×100); **(C)** The endometrium in the specimen of a total hysterectomy showed secretory phase changes (magnification: ×100); **(D)** Low-grade ovarian endometrioid carcinoma (magnification: ×200).

During follow-up at approximately 6 months post-hysterectomy, transvaginal ultrasound identified a 47 × 35 mm cystic–solid right ovarian mass categorized as Ovarian-Adnexal Reporting and Data System (O-RADS) ultrasound category 4 (intermediate risk), characterized by papillary projection and vascularized solid component. Pelvic MRI corroborated concern with restricted diffusion in the solid areas and early enhancement on dynamic sequences, consistent with an intermediate-risk O-RADS MRI assessment. Tumor markers remained normal. The patient underwent laparoscopic bilateral oophorectomy with pelvic lymphadenectomy, omentectomy, and peritoneal washings (**Figure 1**). Grossly, the right ovarian capsule was intact; no surface implants identified. Pathology confirmed unilateral low-grade ovarian endometrioid carcinoma with squamous differentiation, histologically similar to the original EC (**Figure 2**). The left ovary, lymphatic tissues, omentum, and peritoneal cytology were negative for malignancy. Immunohistochemistry showed positivity for estrogen receptor (ER), progesterone receptor (PR), CK5/6, wild-type p53, and low proliferative activity (Ki-67, 10%), MMR proteins (MLH1, PMS2, MSH2, MSH6) were retained. Postoperative recovery was uncomplicated, with careful ongoing surveillance. The timeline of the patient’s diagnosis, intervention, and follow-up process is shown in **Table 1**.

**Table 1:** Chronological timeline of diagnosis, intervention, and outcomes.

Time point (approximate)	Clinical phase/event	Key actions & interventions	Findings & outcomes
Months 0	Initial Diagnosis	Patient presents with abnormal uterine bleeding. Transvaginal ultrasound, diagnostic curettage, pelvic MRI.	Diagnosis: Stage IA, Grade 1 Endometrioid EC.
Months 0-2	Fertility-Sparing Therapy (Initiation)	Started high-dose oral MA. Follow-up hysteroscopy and biopsy.	Promising response, but patient developed significant gastrointestinal side effects.
Month 2	Fertility-Sparing Therapy (Modification)	Discontinued oral MA. LNG-IUS placed.	Treatment continued with better tolerance.
Months 6, 12	Treatment Surveillance	Serial endometrial biopsies.	Histologic Remission Achieved: No evidence of atypia or carcinoma.
Month 13	End of Initial Treatment Phase	LNG-IUS removed. Counseling on next steps (pregnancy vs. continued progestin).	Patient in remission.
Month 18	Definitive Uterine Surgery	Patient’s goals changed. Laparoscopic total hysterectomy + bilateral salpingectomy (ovarian preservation).	No Residual Disease: Uterine pathology confirmed secretory endometrium without carcinoma.
Months 18-24	Post-Hysterectomy Surveillance	Regular clinical follow-up including pelvic exams and transvaginal ultrasound.	Ovaries appeared normal on initial scans.
Month 24 (~6 months post-hysterectomy)	Detection of New Abnormality	Routine surveillance ultrasound. Follow-up pelvic MRI.	Suspicious Ovarian Mass: Cystic-solid right ovarian mass (O-RADS 4) detected.
Shortly after Month 24	Ovarian Cancer Diagnosis & Staging	Laparoscopic bilateral oophorectomy, pelvic lymphadenectomy, omentectomy, peritoneal washings.	Diagnosis: Unilateral, low-grade Ovarian Endometrioid Carcinoma. Staging negative.
Present	Ongoing Follow-up	Clinical and radiologic surveillance post-oophorectomy.	No adjuvant therapy administered. Patient remains under surveillance.

EC, endometrial carcinoma; MA, megestrol acetate; LNG-IUS, levonorgestrel-releasing intrauterine system; O-RADS, Ovarian-Adnexal Reporting and Data System.

## 3 Discussion

Standard treatment for endometrial carcinoma traditionally involves hysterectomy with bilateral salpingo-oophorectomy, often with additional lymphadenectomy in selected cases. Fertility-sparing strategies utilizing progestin-based hormonal therapies have emerged as safe and feasible alternatives for carefully selected younger women desiring fertility preservation (2). Common fertility-sparing protocols include high-dose oral progestins (e.g., medroxyprogesterone acetate (MPA) 250–500 mg/day or MA 160–320 mg/day) and/or an LNG-IUS, with endometrial sampling every 3 months until complete response and then every 3–6 months for at least 2 years. Eligibility criteria include stage IA disease, absence of myometrial invasion, no extrauterine disease, and no contraindications to progestin usage (3). Cross-sectional imaging is recommended at baseline to exclude extrauterine disease; during surveillance, imaging is symptom- or finding-driven.

While definitive hysterectomy after childbearing is complete is strongly recommended to minimize recurrence risk, guidance on the optimal long-term management for patients who decline or defer surgery is still evolving (4). Continued conservative uterine management with an LNG-IUS may be acceptable in selected cases where hysterectomy is declined (5). Furthermore, the role of concurrent oophorectomy in young patients (< 45 years old) is controversial given the implications on ovarian endocrine function, quality of life, and bone and cardiovascular health. Recent studies support ovarian conservation as safe and associated with favorable outcomes in carefully selected premenopausal, early-stage EC patients (6). However, careful preoperative imaging and thorough intraoperative exploration are critical. Following hysterectomy with ovarian preservation in low-risk patients, reasonable follow-up includes pelvic examination and transvaginal ultrasound every 6–12 months, reserving MRI for indeterminate or suspicious sonographic findings. Routine serum CA-125 monitoring is generally not indicated unless elevated at baseline.

Despite favorable remission rates with conservative progestin therapies, subsequent ovarian malignancies remain concerning. Literature reports a wide range (approximately 3.6-25%) of ovarian cancer concurrence among young EC patients previously treated conservatively, reflecting variability in criteria, populations, and sample sizes (7–9). Such ovarian lesions are frequently associated with aggressive features (myometrial invasion, lymphovascular invasion, higher tumor grade). Nonetheless, cases of ovarian malignancies manifesting with favorable prognoses in otherwise low-risk EC patients have also been observed.

Occurrence of ovarian carcinoma following successful conservative EC management-particularly subsequent to a clinically definitive hysterectomy-is extremely rare. In a large study (n=319), subsequent extrauterine malignancies were detected in 2.2% of cases, predominantly linked with persistent uterine disease at time of hysterectomy (10). The current case notably featured no identifiable residual uterine malignancy or myometrial invasion at hysterectomy, rendering subsequent ovarian malignancy particularly unusual.

Recent genomic analyses further indicate many synchronous ovarian and endometrial cancers share clonal origins, redefining previous categorizations into synchronous primary versus metastatic disease (11). Considering our patient’s clinical progression, the pathogenesis (primary ovarian neoplasm versus metastatic spread) remains unclear. Histopathological similarity strongly suggests related tumor origin and highlights the possibility that clinically undetected microscopic disease persisted or disseminated prior to hysterectomy. Definitive distinction between metastatic disease and a *de novo* primary would require comparative molecular profiling (e.g., targeted sequencing for shared mutations in PTEN, PIK3CA, CTNNB1, ARID1A, MMR status, or POLE). The absence of such analyses is a limitation of this report.

Our management leveraged a standard high-dose progestin regimen with transition to LNG-IUS for toxicity, paired with 3-month interval histologic assessments—an approach consistent with guidelines. The decision to preserve ovaries at hysterectomy balanced oncologic risk with endocrine and quality-of-life considerations and was supported by normal imaging and low-risk histology; however, this case illustrates the residual risk of occult or metachronous ovarian malignancy. Baseline MMR testing and genetic counseling for Lynch syndrome in a young patient would have strengthened risk assessment; their absence represents a limitation. Post-hysterectomy surveillance prioritized clinical evaluation and ultrasound, reserving MRI for indeterminate findings. In retrospect, our follow-up strategy was appropriate and facilitated early detection, but prospective, standardized surveillance protocols for patients undergoing fertility-sparing followed by ovarian conservation remain needed. Early detection and prompt surgical intervention markedly improve prognosis and survival potential.

## 4 Conclusion

This case illuminates the notable clinical challenge of ovarian malignancies arising subsequent to effective fertility-sparing management and hysterectomy for early-stage EC. Even after achieving clinical remission, the risks of ovarian malignancy remain tangible. Consequently, robust patient education and meticulous postoperative surveillance are imperative. While standardized surveillance protocols are not established, regular clinical follow-up including pelvic examination and transvaginal ultrasound remains a reasonable approach to monitor the preserved ovaries, with a low threshold for further imaging if abnormalities are detected.

## 5 Patient perspective

The patient reported that preserving fertility initially motivated her choice of conservative therapy. After achieving uterine remission, her priorities shifted toward definitive management. She valued shared decision-making and close surveillance, which she felt facilitated early detection and timely treatment of the ovarian lesion. She reflected that preoperative counseling on the small but present risk of subsequent ovarian malignancy helped her recognize symptoms and adhere to follow-up. She consented to share her experience to inform other patients and clinicians.

## 6 Limitation

While the report is valuable for raising vigilance about the risk of ovarian malignancy after conservative management for EC, its findings must be interpreted within the limitations of single-patient experience, absence of genomic analysis, and short follow-up. The absence of paired tumor sequencing to assess clonality between uterine and ovarian lesions precludes definitive classification as metastatic versus *de novo* primary disease. Further research, including molecular profiling and standardized surveillance recommendations, is necessary to guide future management.

## Data Availability

The original contributions presented in the study are included in the article/Supplementary Material. Further inquiries can be directed to the corresponding author.
